# Sex differences in prelimbic cortex calcium dynamics during stress and fear learning

**DOI:** 10.1186/s13293-024-00653-9

**Published:** 2024-10-16

**Authors:** Ignacio Marin-Blasco, Giorgia Vanzo, Joaquin Rusco-Portabella, Lucas Perez-Molina, Leire Romero, Antonio Florido, Raul Andero

**Affiliations:** 1 Institut de Neurociències, Universitat Autònoma de Barcelona, Cerdanyola del Vallès, Barcelona, Spain; 2 Departament de Psicobiologia i de Metodologia de les Ciències de la Salut, Institut de Neurociències, Universitat Autònoma de Barcelona, Barcelona, 08193 Spain; 3 Department of Psychiatry, School of Medicine, University of North Carolina at Chapel Hill, Chapel Hill, NC USA; 4 Department of Applied Physical Sciences, College of Arts and Sciences, University of North Carolina at Chapel Hill, Chapel Hill, NC USA; 5 Centro de Investigación Biomédica En Red en Salud Mental (CIBERSAM), Instituto de Salud Carlos III, Madrid, 28090 Spain; 6 Unitat de Neurociència Traslacional, Parc Taulí Hospital Universitari, Institut d’Investigació i Innovació Parc Taulí (I3PT), Institut de Neurociències, Universitat Autònoma de Barcelona, Bellaterra, 08193 Spain; 7 ICREA, Pg. Lluís Companys 23, Barcelona, Spain

**Keywords:** Sex differences, PFC, Stress, Fear learning, Fear memory, Calcium imaging, Miniscopes, Movement

## Abstract

In recent years, research has progressively increased the importance of considering sex differences in stress and fear memory studies. Many studies have traditionally focused on male subjects, potentially overlooking critical differences with females. Emerging evidence suggests that males and females can exhibit distinct behavioral and neurophysiological responses to stress and fear conditioning. These differences may be attributable to variations in hormone levels, brain structure, and neural circuitry, particularly in regions such as the prefrontal cortex (PFC). In the present study, we explored sex differences in prelimbic cortex (PL) calcium activity in animals submitted to immobilization stress (IMO), fear conditioning (FC), and fear extinction (FE). While no significant sex differences were found in behavioral responses, we did observe differences in several PL calcium activity parameters. To determine whether these results were related to behaviors beyond stress and fear memory, we conducted correlation studies between the movement of the animals and PL activity during IMO and freezing behavior during FC and FE. Our findings revealed a clear correlation between PL calcium activity with movement during stress exposure and freezing behavior, with no sex differences observed in these correlations. These results suggest a significant role for the PL in movement and locomotion, in addition to its involvement in fear-related processes. The inclusion of both female and male subjects is crucial for studies like this to fully understand the role of the PFC and other brain areas in stress and fear responses. Recognizing sex differences enhances our comprehension of brain function and can lead to more personalized and effective approaches in the study and treatment of stress and fear-related conditions.

**Supplementary Information:**

The online version contains supplementary material available at 10.1186/s13293-024-00653-9.

## Abstract (plain language summary)

In recent years, researchers have started paying more attention to the differences between males and females in how they handle stress and remember fearful events. Traditionally, many studies focused mainly on males, which might have missed important differences in females. New findings seem to suggest that males and females can respond differently to stress and fear due to differences in hormone levels, brain structure, and brain circuits, especially in the prefrontal cortex (PFC).

In this study, we looked at how male and female animals’ brains reacted to being restrained, experiencing a strong trauma, and then trying to learn a new fearful memory. While their behaviors didn’t show significant differences between sexes, their brain activities did. We found that the prelimbic area of the brain shows calcium activity linked to the animals’ movements during stress and their freezing behavior during fear-related tests.

These results show that the PL is involved in both movement and fear responses. Including both male and female subjects in such studies is vital to fully understand how the prefrontal cortex and other brain areas work in stress and fear situations. Recognizing these differences helps improve our understanding of brain function and can lead to better, more personalized treatments for stress and fear-related conditions.

**Supplementary Information:**

The online version contains supplementary material available at 10.1186/s13293-024-00653-9.

## Highlights

• Sex differences were found in the animals’ calcium activity in the prelimbic cortex (PL) during IMO. Females showed higher frequency, but lower amplitude of calcium events in early IMO.
• No significant sex or stage differences were found in high movement periods during IMO. However, a positive correlation between calcium activity in the PL, and movement was observed in both sexes.
• Both sexes showed increased freezing during fear conditioning (FC). Sex differences were noted during the second extinction session (FE2), with males showing significant changes across tones.
• A negative correlation between calcium activity in the PL and freezing behavior was identified during FC and both extinction phases (FE1 and FE2).
• Significant sex differences in the percentage of excited neurons were observed during FC, but not in inhibited neurons. Differences were also noted in the percentage of excited neurons during FE1, but not during FE2.

**Supplementary Information:**

The online version contains supplementary material available at 10.1186/s13293-024-00653-9.

## Introduction

Stress-related disorders, such as generalized anxiety, depression, or post-traumatic stress disorder (PTSD) have a clear differential prevalence in women and men that can be explained from different points of view, including biological, psychological, genetic, and socioeconomic reasons [1–3]. PTSD is a chronic disease that stems from a response to a high-intensity, negative valence, traumatic experience in vulnerable individuals. Women present a higher prevalence of PTSD, but PTSD-like models in rodents are usually employed only in males [4], thus ignoring the sex-specific neurobiological substrates.

Fear conditioning (FC) is a form of classical conditioning used in research to evaluate fear learning and fear memory [5]. The acquisition of conditioned fear is evoked by repeated pairings of a neutral stimulus − the conditioned stimulus (CS) − with a naturally aversive one − the unconditioned stimulus (US). After this conditioning session, the neutral stimulus, now conditioned (CS), elicits a conditioned response (CR). One of the most critical behaviors to evaluate fear response is freezing, which is the cessation of all movement except for respiration. Freezing is a well-established behavioral indicator of fear in rodents, reflecting the learned association between the CS and the US. In the case of classical conditioning, if the CS is repeatedly presented without the US, a new memory is formed, gradually inhibiting the fear response. Thus, memory systems can induce this updating of previously generated associations, a process known as fear extinction (FE) learning [6].

A key feature of PTSD patients is an impaired extinction of learned fear [7, 8] which plays an important role in the development, persistence, and treatment resistance of clinical symptoms [9]. We have previously shown that exposure to a severe stressor such as immobilization on boards (IMO) presents similar alterations in fear processing, including FE impairments in both male and female mice, as observed in PTSD patients [10, 11].

Fear extinction involves the recruitment of different brain regions, including the prefrontal cortex (PFC), the amygdala, and the hippocampus. The medial PFC (mPFC) is a key region in the regulation of fear learning and memory, by playing distinct roles during the encoding, recall, reconsolidation, and extinction of fear memories [12]. The prelimbic cortex (PL) is a key subregion of the mPFC especially involved in fear conditioning [13]. PL silencing during FE leads to faster within-session decreases in freezing in male rats. This enhanced learning is not sustained in future extinction sessions without PL silencing [14]. Further evidence derives from a study where animals exhibiting extinction impairment also present increased PL activity during the FC and FE [15]. These results suggest that although the PL promotes the expression of conditioned fear it is not crucially involved in acquiring or updating fear memories. Moreover, bidirectional connections between the mPFC and the amygdala have been proven to regulate the conditioning, expression, and extinction of fear [16]. On the other hand, studies focusing on PL activity have rarely considered the potential effects of sex as a biological variable. However, there is evidence suggesting that PL function on fear exhibits a degree of sexual dimorphism. For instance, PL activity in males, but not females, is globally decreased throughout fear extinction recall, and only females exhibited fear extinction-associated increases in PL network excitability [17].

The study of calcium activity during stress and fear procedures taking sex differences into consideration has not been addressed yet. Several studies have used one or two-photon calcium imaging techniques combined with traumatic stress or fear learning procedures mainly in males or not taking into consideration sex differences [18–20]. Thus, studying fear-related processes in both sexes, alongside their neuronal activity correlates in the PL, can help us better understand and delineate the role of the PFC in fear learning [21]. Therefore, we here explore PL calcium activity during IMO, FC, and FE in both male and female mice. We employed behavioral and neuronal recordings using one-photon calcium imaging techniques (UCLA Miniscope v3.1) in freely moving animals. To our knowledge, this experiment entails the longest calcium imaging recordings in freely behaving animals during a FC and FE protocol considering sex differences. We also developed a computational pipeline for comparative Miniscope data analyses.

## Methods

### Animals

We used 7 male and 7 female C57BL/6J adult mice which were 8 weeks old at the beginning of the experiments. Animals were grouped and housed in a temperature-controlled room (23 ± 1 C) under a 12:12 light: dark cycle and food and water were provided ad libitum throughout the procedure. All animals were bred at the Animal Facility at the Institut de Neurociències at the Universitat Autònoma de Barcelona. All procedures were performed between 9 am and 4 pm. Experimental protocols were performed according to the European Community Council Directive 2010-63-UE and the Spanish RD 53/2013 and approved by Generalitat de Catalunya and the Autonomous University of Barcelona (CEEAH-UAB 12033).

### Stereotaxic surgery and histological validation

#### Viral injections

**Fig. 1:**
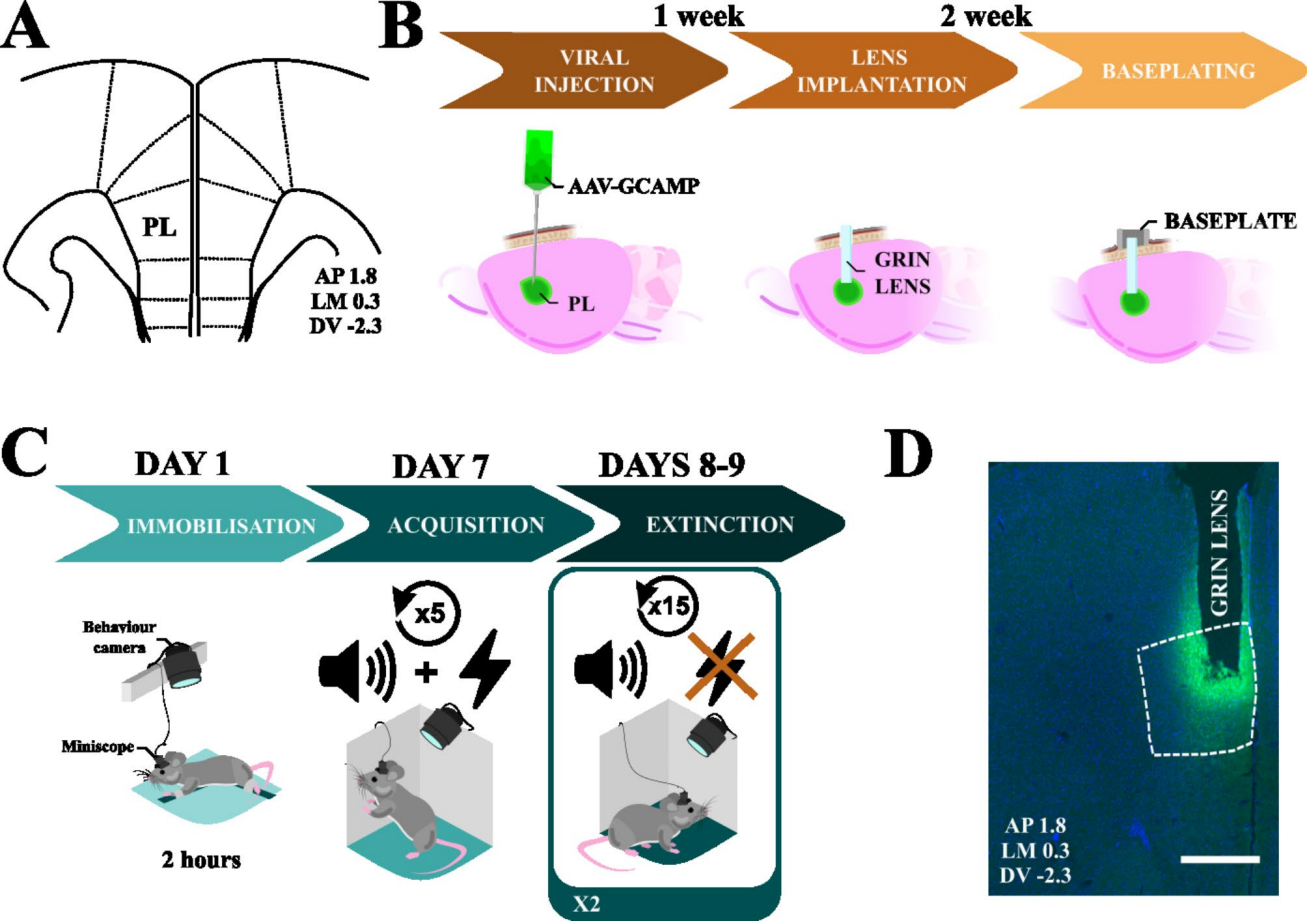
Surgical and behavioral procedures. **(A)** Anatomical location of the mouse PL cortex as described by Paxinos and Franklin’s Mouse Brain Atlas (2008) in stereotaxic coordinates [22]. **(B)** Summarizes the surgical procedures. **(C)** Summarizes the behavioral section of the experiment, starting with the immobilization, the cued-fear conditioning (FC, CS + US), and fear extinction (FE1 and FE2, CS but no US). **(D)** Shows an example of the histological validation of lens placement in the PL cortex. The scale bar corresponds to 500 μm

Animals were anesthetized with isoflurane (IsoFlo, Proima Ganadera SL, Spain) in oxygen at 4% for induction, and 2.0–2.5% for maintenance, with an airflow of 1.25 L/min. After placing the animal in the stereotaxic frame (Kopf Model 962, Harvard-Panlab, Barcelona, Spain), the skull was exposed and cleaned using saline serum (0.9% NaCl). The skulls of the animals were correctly placed in the stereotaxic frame according to bregma and lambda. Once the coordinates (AP + 1.8 mm, LM ± 0.3 mm, DV − 2.3 mm, Fig. 1A) were identified, the skull was drilled, and animals were injected with 800 nL of AAV expressing GCaMP6f calcium indicator (AAV1/2-Syn-WPRE-SV40-GCaMP6f; Addgene, USA) produced at UAB-Viral Vector Production Unit (UPV-UAB, Bellaterra, Spain). Infusions were performed at a speed of 0.07 µL/min using a Hamilton syringe (75RN model; Cibertec-Harvard, Madrid, Spain) coupled to an injection pump (KD Scientific, MA, USA). After the injection, the syringe was removed ensuring that no liquid refluxed from the injection site. Animals were then sutured and left to rest under close supervision for one week (Fig. 1B).

#### GRIN lens implantation and baseplating

After the one-week recovery, animals were anesthetized and head-fixed to the stereotaxic frame. After exposing the skull, a 0.5 mm-tip drill was used to perforate the bone at the previously mentioned AP and LM coordinates (AP + 1.8 mm, LM ± 0.3 mm). Then, a guide hole was made in the brain using the stereotaxic device and a 0.5 × 16 mm blunt needle, reaching a depth of 100 μm above the viral injection site (DV -2.2 mm). Then, a 1.0 pitch, 0.5 mm-diameter, 4 mm-length GRIN lens (Inscopix, CA, USA), was implanted exactly at the same AAV injection site (DV -2.3 mm). Throughout this process, saline serum was constantly applied to the entry point as a lubricant. After complete insertion, the lenses were fixed using cyanoacrylate adhesive. The exposed scalps were protected with dental cement (Rebaron, GC Dental) and stained with black ink (Edding T-100). The exposed sections of the lenses were protected by covering them with plastic caps (0.2 mL PCR Eppendorf tube caps) fixed with fast adhesive. Animals were then individualized to prevent complications over the implants derived from social contact and monitored during a 15 days-recovery. (Fig. 1B). A mouse brain atlas image showing AAV vector spread and GRIN lens placement in the PL region was included in supplementary material (Fig. S1). After two weeks of post-operative care, the protective caps were removed, and a metallic baseplate was attached to the skull using stained dental cement. The calcium signal was simultaneously monitored with the Miniscope to ensure the optimal optical focus for each animal (Fig. 1B). Animals were then left to rest for one week before undergoing the stress and behavioral treatments.

### IMO, fear conditioning and fear extinction

#### General considerations

PL calcium activity and behavior of animals were simultaneously recorded during IMO using the Miniscope system and a 2MP USB Camera with a 3.7 mm pinhole lens (Zhejiang AILIPU Technology, China). During the FC and FE paradigms, the freezing behavior of animals was registered using the StartFear Combined system and the Freezing v1.3.04 software (Harvard-Panlab, Barcelona, Spain).

Previously to IMO exposure, animals underwent three sessions of habituation to the fear cages and the Miniscope for three consecutive days (one session per day). These habituations consisted of 10 min of individual free exploration carrying the Miniscope. The same context was used for habituation and FC (context A) consisting of a grid floor (25 bars of 3 mm in diameter and 10 mm spacing), yellow light, and ethanol solution (70% v/v) for cleaning. The context used for FE (context B) consisted of a continuous metallic floor, a red-light source, and a CR-36 cleaning solution (Jose Collado SA, Spain). Animals were left in their housing enclosure 5 min before and after FC and FE to aminorate the stress caused by the Miniscope placement on the head of the animals.

## Immobilization stress

On the three days following the last habituation session, animals were exposed to three different manipulations to habituate them for the IMO. These manipulations were given once per day. First, animals were exposed for 20 min to a new environment consisting of a box with an area of ​​25 cm^2^ for free exploration. The following day, animals were introduced inside a 50 mL Falcon tube, with a drilled cap to permit air transpiration and facilitate breathing [23]. The day after these habituation sessions, animals were exposed to IMO for 2 hours. The IMO procedure was conducted in a room separated from housing and behavioral tests. Each animal was immobilized by gently attaching their four limbs in a prone position to metal arms attached to a plastic board restricting their movement, as previously described [11]. Recordings of calcium activity and behavior were taken for the first and the last 10 min of the IMO procedure. After the exposure to IMO, animals were left undisturbed for six days until the FC session.

### Fear conditioning and extinction

The protocol used for FC six days after IMO was performed in context A and consisted of a 5-minute intra-session habituation; five 30-second-long tones (30s, 6 kHz, 75 dB) coterminated with an electric footshock (1s, 0.3 mA), and separated by 180-second intertrial intervals (ITI); and 180 final seconds after the last CS-US pairing. The two following days, animals underwent two FE sessions (one session per day), performed in context B. Each FE session consisted of a 5-minute intra-session habituation followed by 15 uncoupled (no shock) 30-second-long tones (30s, 6 kHz, 75 dB), spaced by 30-second ITI (Fig. 1C), finishing with 30 s after the last tone.

### Calcium imaging recording and analysis

#### General considerations

Throughout sessions, calcium activity (GCaMP6f 488 nm fluorescence) was registered using a Miniscope (v3.2 UCLA University, CA, USA) coupled to the Miniscope DAQ hardware and software. The Miniscope system is an open-source microscopy platform for recording and analyzing neural activity in freely behaving animals, developed by Daniel Aharoni Lab (UCLA University, CA, USA).

The Miniscope relied on a 0.25 pitch, 2 mm-diameter GRIN lens (Inscopix, CA, USA), coated with black heat shrink tubing. The Miniscope DAQ software was set up with a recording rate of 30 frames per second. The LED intensity and digital gain were adjusted for each animal and kept constant throughout the experiment [24].

For FC, data were obtained from 5 females and 6 males; for FE1, we have 5 females and 6 males; and for FE2, there are 5 females and 6 males for all calcium imaging recordings. Exclusion from one session was due to outlier removal or recording issues. It did not lead to the removal of the animals from all tasks given that the experiment was performed on all animals using the same conditions. However, animals exhibiting recording issues either at the start or end of the IMO were excluded from all IMO analyses, leaving 5 males and 5 females in this task.

#### Calcium signal deconvolution

**Fig. 2:**
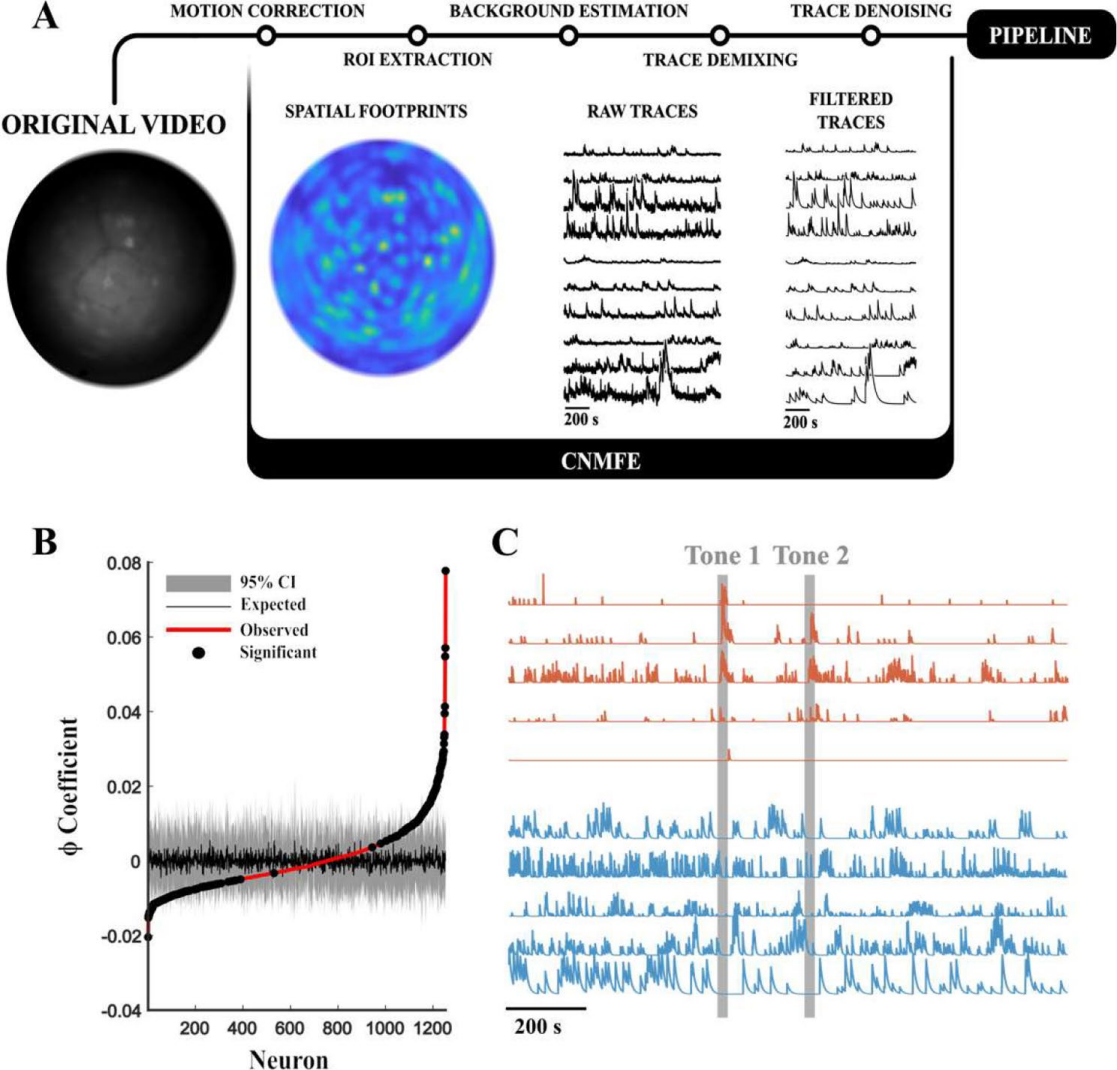
Computational methods for the calcium imaging videos. **(A)** shows the overall working pipeline of CNMF-E from the original video until the extraction of filtered traces. From left to right are shown an unprocessed frame from an IMO session, the spatial footprints for the set session, and the raw and filtered traces for the first 15 identified ROIs. **(B)** shows, for a single session, the average shuffled ϕ coefficient for all the neurons in a single session with their 95 Confidence Interval (CI). In red are shown the ϕ scores for the original traces. Dots label all neurons exhibiting a ϕ score outside the 95CI range. **(C)** shows sample stimulus-excited (red) and stimulus-inhibited traces (blue)

We used the publicly available algorithm, Constrained Non-Negative Matrix Factorisation optimized for Endoscope signals (CNMF-E), to deconvolve the calcium signal [25]. CNMF-E first identifies non-linear shifts in the visual field using an optimized template-matching algorithm, NoRMCorre [26]. All data are reconstructed using the extracted shift vectors to erase motion artifacts. Following this motion correction, the visual field is segmented into regions of interest (ROI) using the CNMF-E algorithm. This delineates the spatial footprints of single-neuron somas. By measuring the fluorescence pattern of different ROI, CNMF-E can identify and discriminate different, yet overlapping, neurons and demix their signal where they overlap (“Trace demixing”, Fig. 2). Lastly, each ROI’s fluorescence trace is fit by a spike-decay model of the calcium imaging indicator and transformed to express fluorescence as a relative change in fluorescence, $\:\varDelta\:F$ (Fig. 2A).

For all sessions, the following set-up was used: spatial downsampling = 3; motion correction = non-rigid; temporal downsampling = 3; dendrite identification = false; spatial algorithm = hals; include residuals = false; deconvolution method = foopsi, minimum spike size = 5 x noise; de-trend method = spline; background model = svd.

For fear conditioning (FC) and fear extinction (FE1 and FE2), all single-neuron traces were obtained by the deconvolution step. Furthermore, they were averaged and expressed as a scaled difference from baseline fluorescence levels for whole-field analyses ($\:\varDelta\:F/{F}_{0}$), where $\:{F}_{0}$ is the median of the complete fluorescence, and $\:\varDelta\:F$ is the difference between $\:{F}_{0}$ and the fluorescence trace. We chose the median as the baseline given the vulnerability of the mean to transient changes in the signal. This procedure was carried out to extract, from the values of fluorescence in the tones, the global fluorescence baseline. The goal was to compare data in the tones without biases. For IMO, this step was not necessary, due to the stressful nature of the procedure, which was constant throughout the two hours of the process. Hence, it was possible to analyze fluorescence traces without impactful biases from external inputs (ex.: the shock, or the sound of the tone), which make it necessary to apply the previously described normalization step.

#### PL calcium activity during IMO

The movement of the animal is the main behavior that can be registered while it is immobilized. This movement can be interpreted as the attempt or struggle of the animal to free itself from the IMO. We quantified the movement of the animals as the frequency and the length of high-activity epochs common during the IMO while simultaneously recording PL calcium activity with the Miniscopes. We developed a MatLab code to compute global movements within the field of vision. All RGB frames were converted to grayscale, and edges were extracted using the Sobel edge detection method (built-in MATLAB^®^ function). Thresholds for edge detection were extracted on a frame-to-frame basis using the canny method [27] and scaled by a fudge factor of 0.5. This allowed us to reduce the amount of data processed by avoiding taking into consideration the entirety of the animal. This edge detection returned, for each frame, a binary edge mask. We expressed this mask as a width $\:\times\:\:$ height matrix. The movement was characterized as the percentage of pixels at *t = n*, that were not present in *t = n−1*. These scores were divided by the count of total edge-bearing pixels identified, *t = n*. Using test videos, we validated that this measure is a practical approach to detect movement at the field level. Given that trial-to-trial changes in camera position, environment, and mouse dimensions can impact this variable, all movement traces were shifted to a median equal to 0 and scaled by the 95% confidence interval.

The movement score was used to identify periods of high activity of the animal during the IMO. By measuring the movement in the video field, we found that their distribution can be best described by two distinct normal distributions. These correlate to low and high movement frames. We defined the intersection between the two distributions as the classifier to select high-movement frames. For each animal, we calculated the number of frames that animals spend in this high-movement status. With this method, we analyzed differences in the percentage of high-movement events, in their length and frequency.

Furthermore, we decided to test calcium activity and movement for correlation, employing Pearson’s Test. First, we analyzed the data globally, by testing movement, during early and late IMO, with the global fluorescence activity in both females and males (as two different groups). Secondly, we decided to look into individual neurons and identify the specific percentages of correlated (positively and negatively) and uncorrelated neurons. We use Pearson’s correlation test between individual neuronal fluorescence traces and movement scores for each animal in early and late IMO. We defined neurons as uncorrelated with *p* > 0.05, positively correlated with *p* < 0.05 and R factor > 0, and negatively correlated with *p* < 0.05 and R factor < 0. We divided the neurons into two groups, belonging to females and males, and the analysis was done for early and late IMO.

#### Fear memory, freezing, and PL calcium activity

For FC and FE sessions, behavioral video data was obtained with the Freezing v1.3.04 software (Harvard-Panlab-Barcelona, Spain), with a resolution of one immobility measure per second (expressed in %). Data were binarized as freezing or non-freezing. The first is regarded as a percentage of immobility beyond a respiratory rate above 0.7.

We decided to test calcium activity and freezing for correlation, employing Pearson’s Test. First, we analyzed the data globally, by testing global fluorescence and freezing during a specific session (FC, FE1, and FE2) in both females and males (as two different groups). Secondly, we decided to look into individual neurons and identify the specific percentages of correlated (positively and negatively) and uncorrelated neurons. We use Pearson’s correlation test between individual neuronal fluorescence traces and freezing for each animal in FC, FE1, and FE2. We defined neurons as uncorrelated with a *p* > 0.05 significance, positively correlated with *p* < 0.05 and R factor > 0, and negatively correlated with *p* < 0.05 and R factor < 0. We divided the neurons into two groups, belonging to females and males, and the analysis was done for all sessions (FC, FE1, and FE2).

#### Identifying stimulus-responding neurons

Current methods for estimating neuronal activity in fear memory with Miniscopes often rely on global-field measurements. However, this approach can result in the simplification of a population with heterogeneous neuronal responses. To address this issue, we developed a different method to compute the modulatory effects of the different tones on calcium dynamics. Firstly, we defined a calcium event as a local maximum in the filtered calcium trace. Following, each neuron’s activity was expressed as a Boolean (true or false) indicating the temporal location of calcium events. For each neuron, the Phi Coefficient (ϕ; a measure of association for two binary variables), was computed to describe the association between tone presentation and calcium responses. To this purpose, we define “true positive” as a calcium event during tone presentation and “false negative” as a frame without a calcium event during tone presentation.

To avoid delimiting a global ϕ threshold, all event matrices were randomized, and ϕ scores were computed 1000 times. Results were stored in a matrix, *R.* Original scores were expressed as Z distance from the population matrix R. Z distance (or absolute Z-score) is a statistical measure that quantifies the distance between a data point and the mean of a dataset.

All formulas used were included in Supplementary Material.

### Histological processing

After termination of the protocols, animals were anesthetized with Isoflurane, perfused first with saline serum (0.9% NaCl) and afterwards with 4% paraformaldehide (Casa Alvarez, Spain) for later histological processing and validation of AAV injection and lens placement (Fig. 1D, Fig. S1). Brains were extracted, cryopreserved in 30% Sucrose and frozen in dry ice-cooled (-55^o^C) isopentane (Sigma-Aldrich). Then, 30 μm coronal sections of the PL were obtained using a cryostat (Leica CM3050). Sections were then stained with DAPI (1:20000), and mounted on Superfrost slides (Thermo Scientific, USA). Next, a fluorescence microscope (Nikon Eclipse 90i) was employed to capture images depicting the distribution of the vector-associated reporter (EGFP). These images were then overlapped with the DAPI image (Fig. 1D).

### Statistical analyses

We used MATLAB code in the R environment including the following tests: Shapiro-Wilk for testing for violations of normality within groups and across the global sample. Levene’s tests for equality of variances. Grubb’s recurrent test for outlier identification. One-way and repeated measures ANOVA for testing for differences between and within groups. We used Tukey’s test for multiple comparison analysis. Furthermore, to test for a correlation between calcium fluorescence and either freezing or motion, we used the Pearson’s Correlation Test.

Statistical analyses were integrated with the MATLAB code and tailored to the specific data tested. All codes (MATLAB and R) of the complete pipeline and all the hyperparameters are available on our *GitHub*.

## Results

### PL calcium activity during IMO shows sex differences

**Fig. 3:**
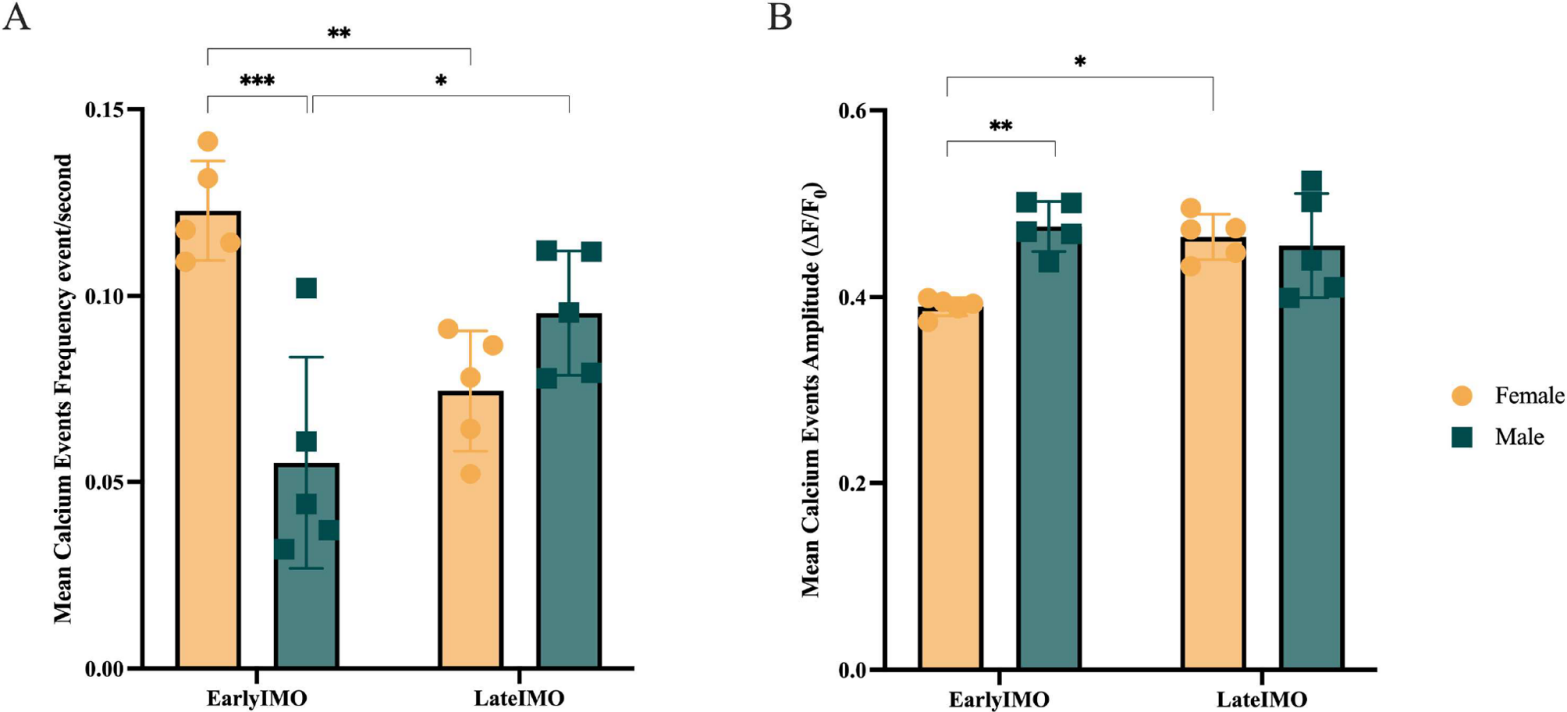
Sex differences in PL calcium activity (global fluorescence) during early and late IMO. Mean calcium fluorescence events’ frequency (events/second) **(A)** and amplitude (ΔF/F0) **(B)** for the first (early) and last (late) ten minutes of the 2 h of exposure to IMO, in yellow, females, and in green, males. Single-animal mean values are shown as dots in each plot. Results are expressed as means ± Standard Error of the Mean (SEM), *n* = 5 for both sexes. Differences were tested using repeated measures ANOVA after checking for normality, sphericity, and removing outliers with the Grubbs’ test (* *p* < 0.05, **p < 0.01, ***p < 0.001)

We sought to understand how the PL responds to this high-intensity stressful stimulus and whether PL activity during the IMO is sex-dependent. We measured the calcium activity during the first and final 10 min of the IMO (early and late IMO, respectively). During early IMO, the frequency of mean calcium fluorescence events was found to be significantly elevated in females compared to males (repeated measures ANOVA, Sex*Epoch F (1,8) = 35.8, *p* < 0.001; Tukey’s Early IMO *p* < 0.001; Fig. 3A). Furthermore, mean calcium fluorescence events’ frequency in females exhibited a clear decrease between the early and late IMO (Tukey’s Early*Late in Females *p* = 0.0017; Fig. 3A). The opposite was observed in male mice (Tukey’s Early*Late in Males *p* = 0.005; Fig. 3A). The mean calcium fluorescence events’ amplitude resulted significantly lower in females than in males in the early IMO (repeated measures ANOVA, Sex*Epoch F (1,8) = 16.21, *p* = 0.0038; Fig. 3B). Females present an increase in mean calcium fluorescence events’ amplitude between early and late IMO (Tukey’s p = 0.0171; Fig. 3B). Males do not show differences between both stages.

### PL calcium activity is coupled to the movement during IMO

**Fig. 4:**
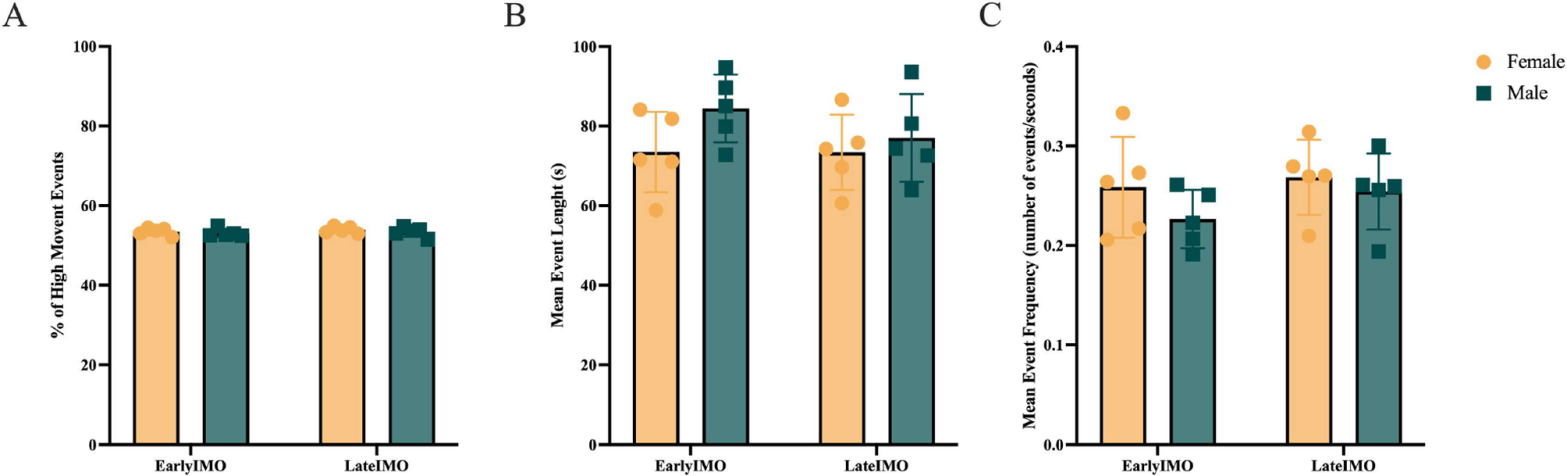
High movement events analysis **(A)** represents the percentage of frames the animals spent in high movement during early and late IMO. **(B)** and **(C)** represent the mean length (seconds) and frequency (events over seconds), respectively, of high movement events during early and late IMO, in yellow, females, and in green, males. Results are shown as means ± SEM, *n* = 5 for both sexes. Differences were tested using repeated measures ANOVA after checking for normality, sphericity, and removing outliers with the Grubbs’ test

We aimed to analyze if changes in PL calcium activity are related to the movement of the animals throughout the IMO. We first evaluated behavioral differences during the IMO by measuring high movement periods in early and late IMO video recordings. We did not find significant differences between sexes nor stages of IMO (early vs. late IMO) in the percentage of frames spent in high movement (repeated measures ANOVA, Sex F (1,8) = 0.595, *p* = 0.463; Epoch F (1,8) = 1.162, *p* = 0.312; Sex*Epoch F (1,8) = 0.029, *p* = 0.870; Fig. 4A). To further analyze and explore differences in the animals’ movement we looked into both the length and frequency of these high movement events (Fig. 4B and C). No differences between sexes or stages of IMO (early vs. late) were encountered in none of the variables [repeated measures ANOVA for Events’ Length (Sex F (1,8) = 2.054, *p* = 0.189; Epoch F (1,8) = 1.092, *p* = 0.326; Sex*Epoch F (1,8) = 1.049, *p* = 0.335; Fig. 4B) and Events’ Frequency (Sex F(1.8) = 1.197, *p* = 0.305; Epoch F (1,8) = 1.992, *p* = 0.196; Sex*Epoch F (1,8) = 0.435, *p* = 0.528; Fig. 4C)].

**Fig. 5:**
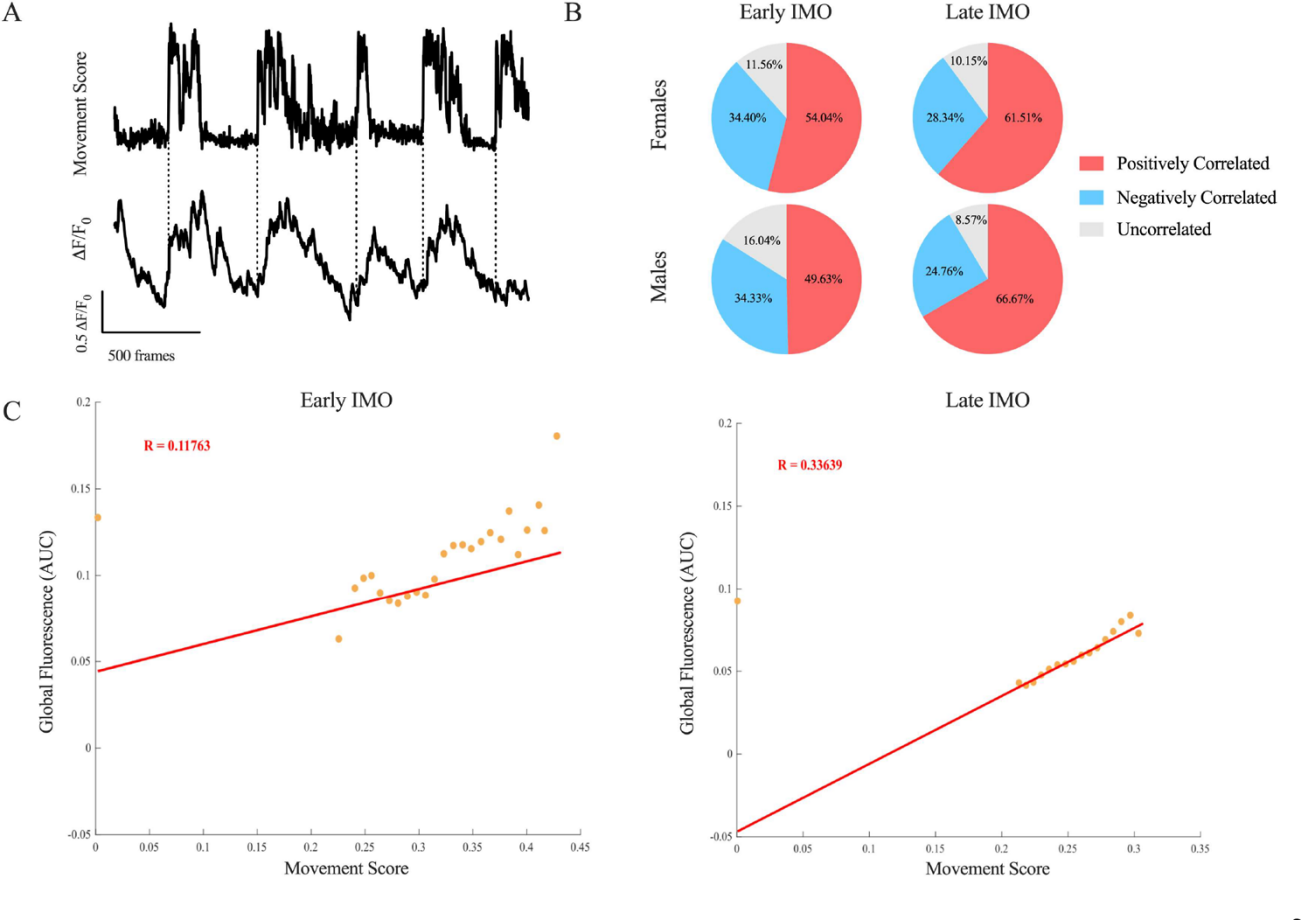
Correlation analysis between PL calcium activity (global and single unit fluorescence) and movement during the IMO **(A)** movement profile and the associated global fluorescence trace of one representing animal during a single early IMO session. Dotted lines represent the onset of a high-movement event. **(B)** results (% of total registered neurons) of Pearson’s Correlation Test between the individual neuron fluorescence traces and the movement of each animal. On the left early IMO and the right late IMO. Number of neurons for females: 718 in early IMO, and 808 in late IMO. Number of neurons for males: 667 for early IMO, and 525 for late IMO. **(C)** Scatter plot of Pearson’s Correlation Test of one representing animal in early (left) and late IMO (right). On the y-axis is the global fluorescence variable (AUC) and on the x-axis is the movement score. Datapoints are displayed binned (bin number = 50). The red line shows the best-fitted model. The R factor of the Pearson’s Correlation Test is also indicated in red

Subsequently, we investigated whether PL calcium activity is related to the movement of the animals during the IMO. The Pearson’s Correlation Test, for each animal, in both early and late IMO, revealed a positive correlation between the global fluorescence of each animal and movement score for both sexes (Fig. 5A and C). Individual data of the animals are shown in Supplementary Table 1.

To further analyze this correlation, we decided to investigate it at single neuron level by exploring the percentages of positively correlated (R factor > 0, *p* < 0.05), negatively correlated (R factor < 0, *p* < 0.05), and uncorrelated neurons (*p* > 0.05) (Fig. 5B). The results show that more than 50% of the registered neuronal population display a positive correlation between fluorescence and movement score for both early (females, 54.04%, and males, 49.63%) and late IMO (females, 61.51%, and males, 66,67%), for each sex. The second-in-size population comprises negatively correlated neurons (early IMO; in females, 11.56%, and males, 16.04%; and late IMO, for females, 10.15%, and males, 8.57%). Uncorrelated neurons are a minority of the neuronal population (early IMO; in females, 34.40%, and males, 34.33%; and late IMO, for females, 28.34%, and males, 24.76%).

### PL calcium activity during fear conditioning and extinction is sex-dependent

**Fig. 6:**
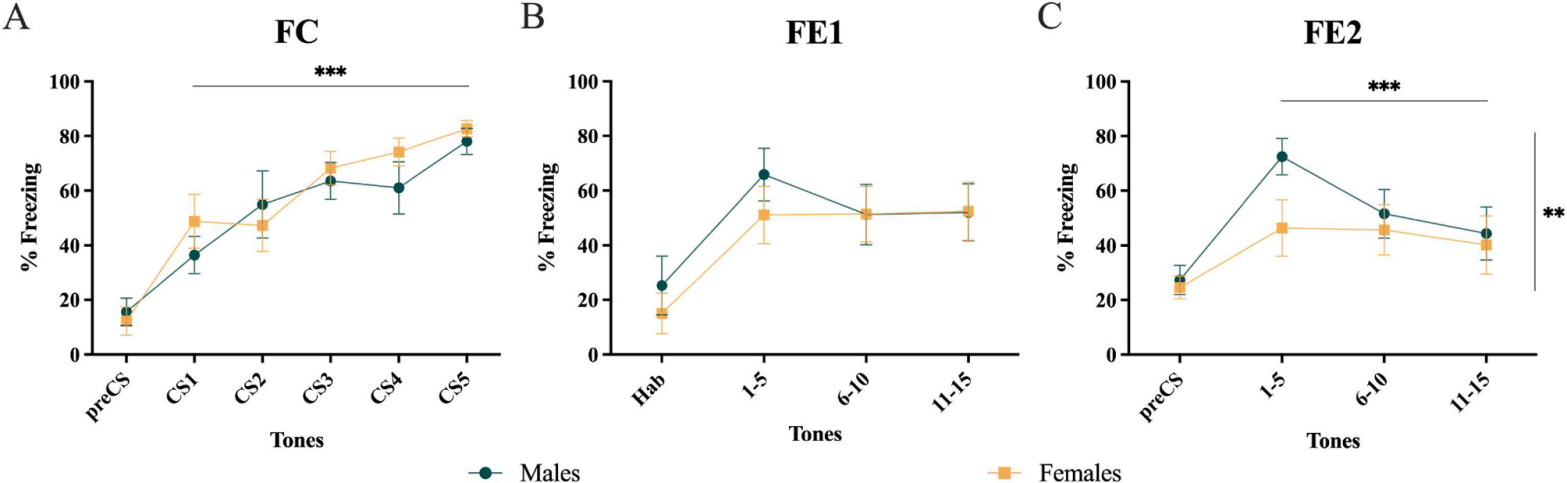
Behavioral sex differences across the fear conditioning and fear extinction paradigms. **(A)** mean tone-inducing freezing score (percentage of freezing time during the tone) for males (green) and females (yellow) across fear conditioning (FC), **(B)** fear extinction session 1 (FE1), and **(C)** fear extinction session 2 (FE2). For FE1 and FE2, freezing scores were grouped and averaged for the first (1,2,3,4,5), middle (6,7,8,9,10), and last (11,12,13,14,15) five tones. Shown are means ± SEM, *n* = 7 for both sexes. Differences were tested using repeated measures ANOVA after checking for normality, sphericity, and removing outliers with the Grubbs’ test (**p < 0.01, ***p < 0.001)

We first sought to determine whether males and females exposed to IMO exhibit differences in freezing during FC and FE. Both males and females exhibited a marked increase in freezing throughout FC during tone (CS) presentations (repeated measures ANOVA, Tones F(4,48) = 0.8, p *<* 0.001; Fig. 6A), thus suggesting an adequate fear acquisition. No significant differences were found in FE1 (repeated measures ANOVA, Tones*Sex F (2,24) = 2.4, *p* = 0.11; Tones F (2,24) = 1.9, *p* = 0.16; SexF (1,12) = 0.1, *p* = 0.69; Fig. 6B). During FE2, we found differences between males and females (repeated measures ANOVA, Sex*Tone F (2,24) = 6.998, *p* = 0.04; Fig. 6C), specifically due to differences in the first five tones (Tukey’s CS (1,2,3,4,5), *p* = 0.02). Also, significant differences were found between the first and the last five tones in males (repeated measures ANOVA, Tones F (1,12) = 14.17, *p <* 0.001; Tukey’s in Males CS (1,2,3,4,5) to CS (6,7,8,9,10), *p* *<* 0.001; and CS (1,2,3,4,5) to CS (11,12,13,14,15), *p <* 0.001; Fig. 6C).

**Fig. 7:**
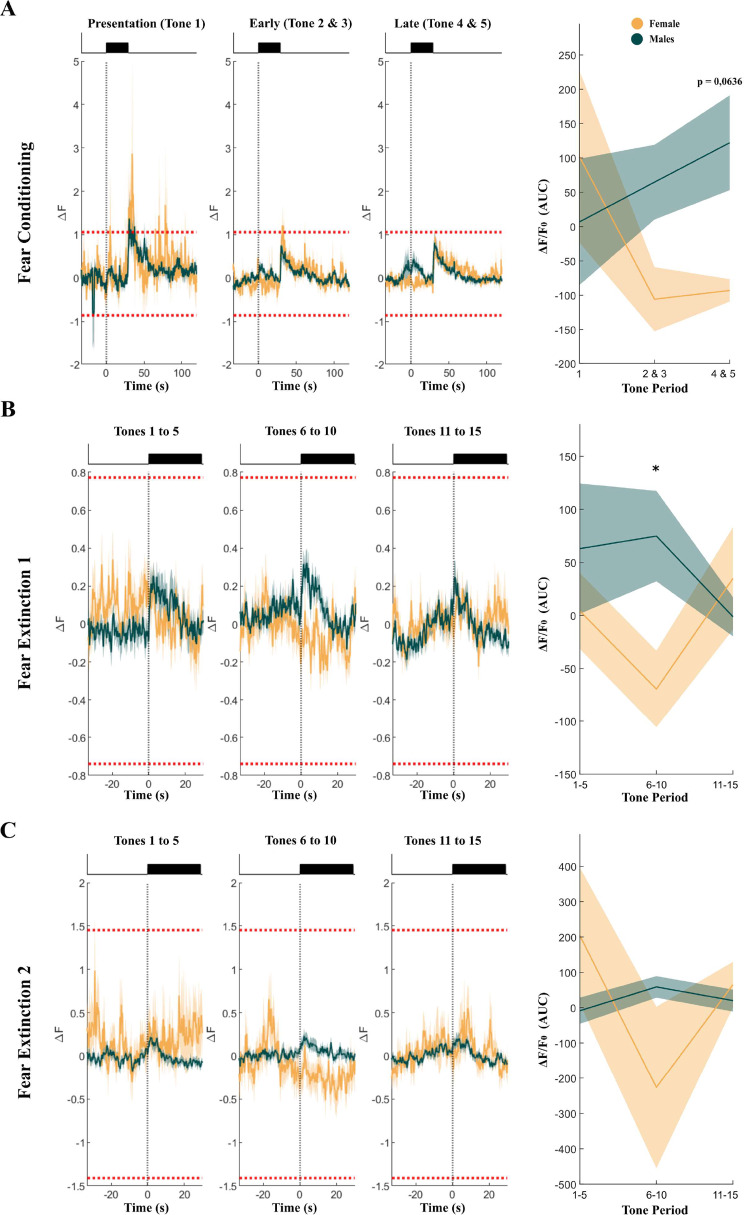
Global fluorescence responses during fear conditioning and extinction. **(A)** on the right shows the mean ± standard deviation (SD) fluorescence responses to the tones (black bars) and shocks throughout the conditioning task. In each graph, red dotted lines show the upper and lower bound for the session. In yellow, females, and in green males. On the left for each animal, the AUC of the fluorescent trace was computed and represented (FC: *n* = 6 for males, *n* = 4 for females). **(B)** to **(C)** on the right represent the global fluorescence for both extinctions, as for FC in A) (FE1: *n* = 6 for males, *n* = 7 for females; FE2: *n* = 7 for males, *n* = 6 for females). Represented data means ± SD. Differences were tested using repeated measures ANOVA after checking for normality, sphericity, and removing outliers with the Grubbs’ test (* *p* < 0.05, **p < 0.01, ***p < 0.001

We subsequently sought to characterize PL activity during FC and FE and explored whether this is sexually dimorphic. We found no significant differences in the global fluorescence of the animals during FC (repeated measures ANOVA, Tones*Sex F (2,16) = 2.313, *p* = 0.131; Tones F (2,16) = 0.462, *p* = 0.638; Sex F (1,8) = 2.279, *p* = 0.169; Fig. 7A and B). No significant differences were found during FE1 (repeated measures ANOVA, Tones*Sex F (2,16) = 1.935, *p* = 0.18; Tones F (2,16) = 0.228, *p* = 0.80; Sex F (1,8) = 3.814, *p* = 0.09; Fig. 7C and D), except for tones 6 to 10 where significant differences between sexes were found (Tukey’s CS(6 to 10), *p =* 0.02; Fig. 7D). No significant differences were found for FE2 (repeated measures ANOVA, Tones*Sex F (2,16) = 1.504, *p* = 0.25; Tones F (2,16) = 0.624, *p* = 0.55; Sex F (1,8) = 0.287, *p* = 0.61; Fig. 7E and F).

**Fig. 8:**
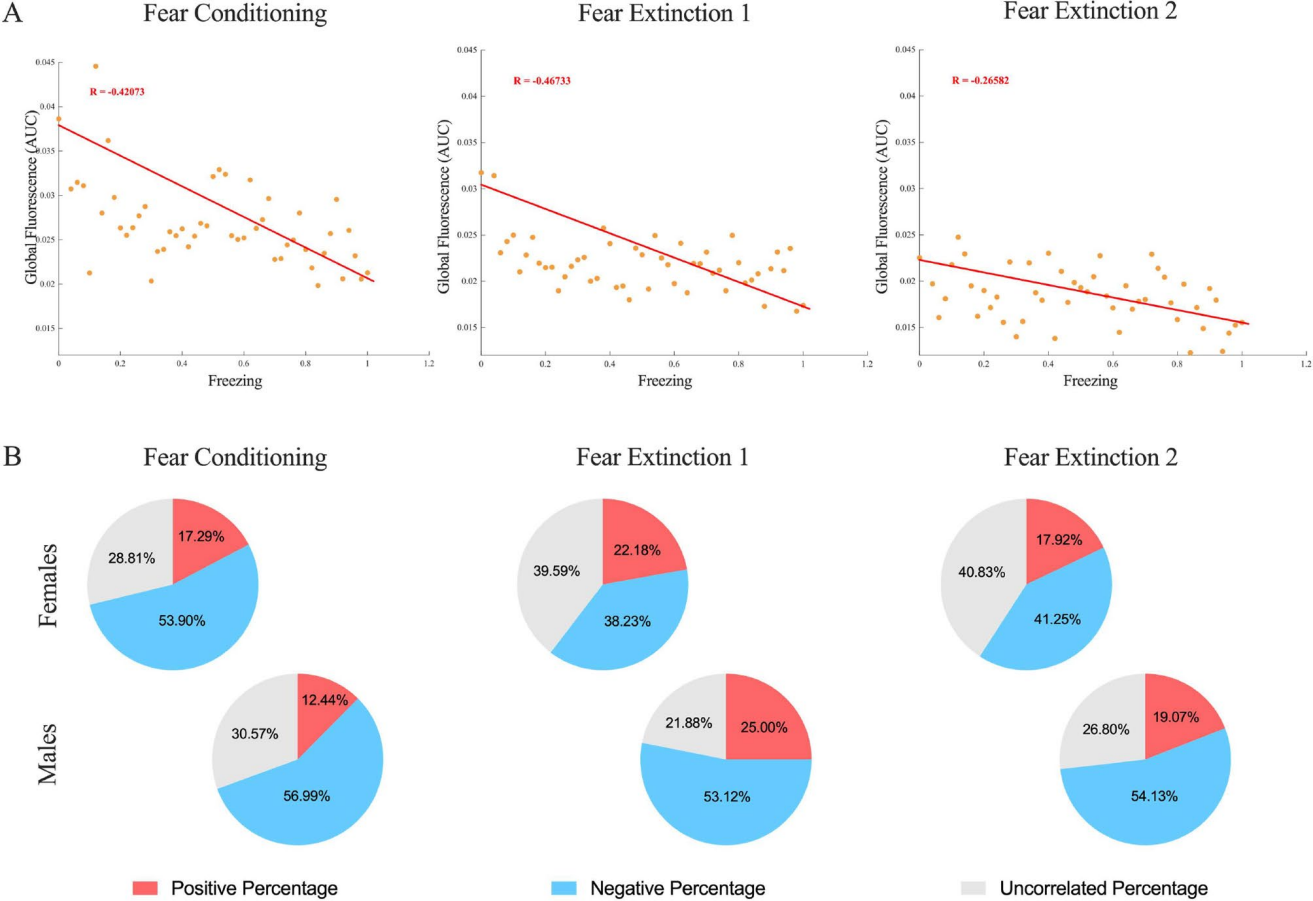
Correlation analysis between PL calcium activity (global and single unit fluorescence) during fear conditioning and extinction. **(A)** scatter plots of Pearson’s Correlation Test for one representing animal in FC, FE1, and FE2. On the y-axis is the global fluorescence variable (AUC) and on the x-axis is the freezing time. Datapoints are displayed binned (bin number = 50). The red line shows the best-fitted model. The R factor of the Pearson’s Correlation Test is also indicated in red. **(B)** results (% of total registered neurons) of Pearson’s Correlation Test between the individual neuron fluorescence traces and freezing time of each animal. From left to right, FC, FE1, and FE2. On the top line females and the bottom males. In red positively correlated neurons, in blue negatively correlated neurons, and in grey uncorrelated neurons (in %)

We subsequently explored the relationship between PL calcium activity and freezing behavior during FC and FE. We used Pearson’s Test to analyze the correlation between global fluorescence and freezing in both sexes during FC, FE1, and FE2. For every stage, the results revealed a negative correlation between the two variables in both sexes (R factor < 0, *p* < 0.001; Fig. 8A, B, and C). Individual data of the animals are shown in Supplementary Table 2.

To further investigate this correlation at single neuron level we determined the percentages of positively correlated (R factor > 0, *p* < 0.05), negatively correlated (R factor < 0, *p* < 0.05), and uncorrelated neurons (*p* > 0.05) (Fig. 8D, E, and F). The results show that more than 50% of the registered neuronal population display a negative correlation between fluorescence and freezing for both FC (56.99%) and FE (FE1, 53.12%; FE2, 54.13%). The second-in-size population comprises uncorrelated neurons for FC and FE2 (FC, 30.57%; FE2, 26.80%), and of positively correlated neurons for FE1 (FE1, 25.00%). Positively correlated neurons are a minority of the neuronal population for FC and FE2 (FC, 12.44%; FE2, 19.07%), while for FE1, the minority is made of uncorrelated neurons (FE1, 21.88%).

### Sex differences in PL excited and inhibited neurons during fear conditioning and extinction

**Fig. 9:**
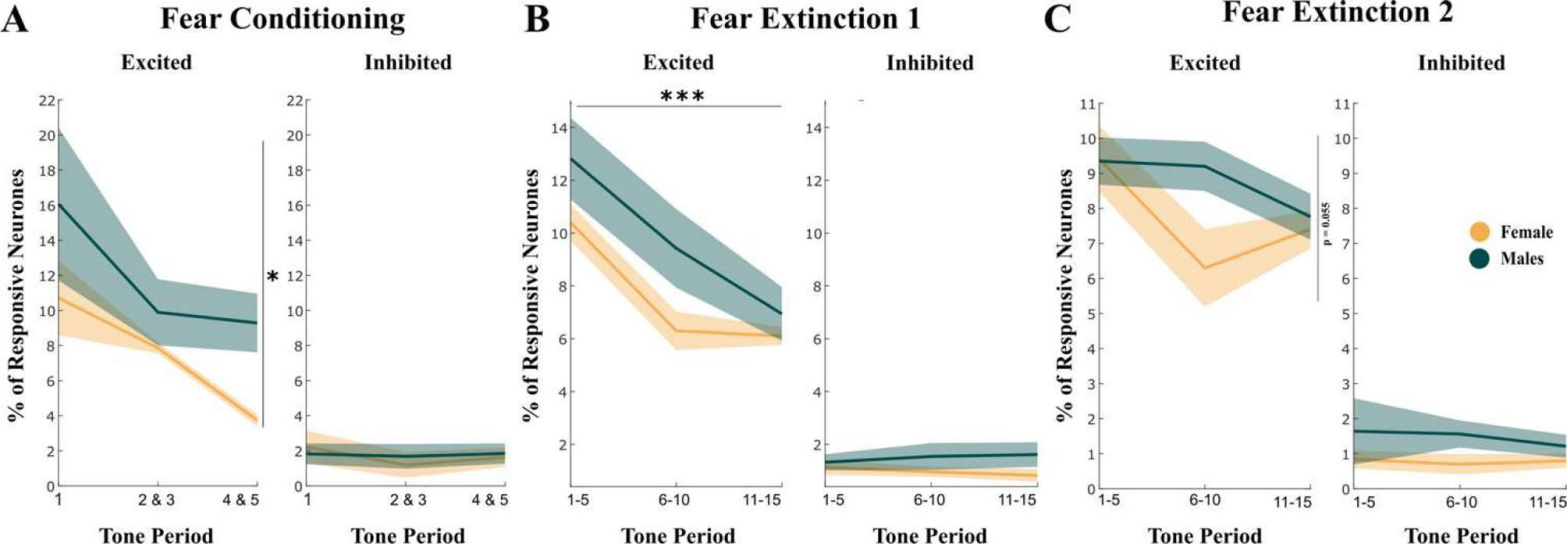
Analysis of excited and inhibited neurons for fear conditioning and extinction. Mean percentage of excited (left) and inhibited (right) neurons to tone (CS) presentation during the conditioning (**A**) and extinctions 1 (**B**) and 2 (**C**). In yellow females, and in green males. Data shown means ± SEM. Differences were tested using repeated measures ANOVA (*p < 0.05, **p < 0.01, ***p < 0.001) (FC: *n* = 6 for males, *n* = 4 for females; FE1: *n* = 6 for males, *n* = 7 for females; FE2: *n* = 7 for males, *n* = 6 for females)

To further analyze PL calcium activity, we identified ratios of excited and inhibited neurons during FC and FE. We found significant differences in the percentage of excited neurons for FC (repeated measures ANOVA, Sex F (1,8) = 6.1217, *p* = 0.0035; Fig. 9A). No significant difference was found for the percentage of inhibited neurons (Fig. 9A). For FE1, we found significant differences for the tones variable (repeated measures ANOVA, Tones F (2,18) = 41.8264, *p* < 0.001; Fig. 9B). No significant differences were noticeable in the percentage of inhibited neurons (Fig. 9B). For FE2, no significant differences were found in either the percentage of excited or inhibited neurons (repeated measures ANOVA, Sex F (1,10) = 6897, *p* = 0.0555; Fig. 9C).

## Discussion

To the best of our knowledge, this is the first study investigating the PL calcium activity correlates of stress and fear learning including females and male individuals. Although we found no relevant sex differences in behavior during IMO, FC, and FE, we did observe them in PL calcium activity using Miniscopes. Specifically, during the early stage of IMO, females exhibited higher frequency and lower amplitude of calcium events than males. Also, females showed a decrease in calcium events’ frequency between early and late IMO, while the opposite was observed in males. Calcium events’ amplitude also increased between early and late IMO, while males maintained the same levels. Regarding FC and FE, females showed lower global fluorescence levels than males during the FE1. To determine if these differences in PL calcium activity were related to processes other than stress and fear-related ones, we conducted correlation studies between the animals’ movement during IMO and freezing behavior during FC and FE. Our results indicated that calcium activity in the PL correlated positively with movement during stress exposure and negatively with freezing behavior during both FC and FE, with no sex differences found in the correlation levels. This suggests that the PL subregion of the PFC might have a significant role in locomotion as well as in fear-related processes.

The observed sex differences in PL calcium activity during IMO and FE could be attributed to several factors, including the influence of sex hormones such as estrogen, which is known to modulate synaptic plasticity and neuronal excitability, potentially explaining the higher frequency and lower amplitude of calcium events in females during early IMO [28, 29]. Additionally, structural and functional differences in the PL and its connectivity with other brain regions involved in stress and fear, such as the amygdala and hippocampus, might underlie these sex-specific patterns of activity [30]. These neural differences could reflect distinct strategies in processing and adapting to stress between males and females, as supported by research showing sex-dependent variability in stress-induced neural activity and subsequent behavioral outcomes [31]. Importantly, the lack of sex differences in the correlation between PL activity and movement or freezing behavior suggests that the observed calcium activity differences are not merely due to movement but likely reflect intrinsic neurobiological differences [7].

Our findings concur with previous studies and highlight the multiple functions of the PFC in fear memory and locomotion. The PL has been shown by previous studies to be crucial for fear memory acquisition and recall with increased firing rates of PL neurons during these processes [32]. Furthermore, calcium imaging experiments have demonstrated the activation of specific neuronal ensembles in the PL during fear memory recall, indicating the significance of coordinated activity in fear retrieval [33]. Besides having an impact on fear memory, the PFC, and especially the PL plays a clear role on locomotion. The firing patterns of PFC neurons correlate with voluntary movement suggesting a role in the planning and initiation of locomotor activity [34]. Moreover, the interaction of the PFC with the motor cortex is important for executing difficult motor tasks as well as adjusting movements according to sensory feedback [35]. Also, locomotion could be influenced by fear conditioning since altered movement patterns following fear conditioning in mice are related to changes in PFC activity [36]. On the other hand, chronic stress-related PFC function impairment can impact both fear responses and locomotor activity, highlighting the connection between emotional processing and motor functions [37]. The correlation between PL calcium activity and both movement during stress and freezing behavior during fear conditioning and extinction underscores the integrated role of the PFC in managing emotional and motor responses. This integration could be crucial for adaptive behaviors, enabling organisms to respond appropriately to threats through both emotional and motor adjustments [38].

Importantly, the sex differences in PL calcium activity observed in our study underscore the importance of including both female and male individuals in stress and fear studies to fully understand the complexity of brain function and behavior. Understanding sex differences in brain function, including the role of the PFC, is crucial for developing comprehensive models of neural mechanisms and for the eventual identification of potential sex-specific therapeutic targets.

## Conclusions

Our results highlight two important aspects in neuroscience studies related to stress, fear, and calcium activity. First, it is essential to include both female and male subjects because, while there may be no differences between sexes in some dependent variables, such as behavior, there can be significant sex differences in other specific parameters such as calcium activity. Second, although the PL plays a crucial role in fear memory and movement, studies in these fields generally do not consider both parameters. Integrating these aspects would benefit the field.

## Electronic supplementary material

Below is the link to the electronic supplementary material.

Supplementary Material 1

## Data Availability

All codes (MATLAB and R) of the complete pipeline and all the hyperparameters and datasets are available on our GitHub (https://github.com/Anderolab/Anderolab). The datasets used and analyzed during the current study are available from the corresponding author upon reasonable request.

## Abbreviations

PFC: Prefrontal cortex
PL: Prelimbic cortex
IMO: Immobilization stress
FC: Fear conditioning
FE: Fear extinction
FE1: First extinction section
FE2: Second extinction section
PTSD: Post-traumatic stress disorder
CS: Conditioned stimulus
US: Unconditioned stimulus
CR: Conditioned response
ROI: Regions of interest

## References

[1] Olff M. Sex and gender differences in post-traumatic stress disorder: an update. Eur J Psychotraumatol. 2017;8:sup4.

[2] Kuehner C. Why is depression more common among women than among men? Lancet Psychiatry. 2017;4(2):146–58. 27856392 10.1016/S2215-0366(16)30263-2

[3] Bangasser DA, Valentino RJ. Sex differences in stress-related Psychiatric disorders: neurobiological perspectives. Front Neuroendocrinol. 2014;35(3):303. 24726661 10.1016/j.yfrne.2014.03.008 PMC4087049

[4] Kaluve AM, Le JT, Graham BM. Female rodents are not more variable than male rodents: a meta-analysis of preclinical studies of fear and anxiety. Neurosci Biobehav Rev, 2022; 143:104962. 10.1016/j.neubiorev.2022.104962 36402227

[5] Maren S. Neurobiology of pavlovian fear conditioning. Annu Rev Neurosci. 2001;24:897–931. 11520922 10.1146/annurev.neuro.24.1.897

[6] LeDoux J. The emotional brain, fear, and the amygdala. Cell Mol Neurobiol. 2003;23(4–5):727–38. 14514027 10.1023/A:1025048802629 PMC11530156

[7] Milad MR, Pitman RK, Ellis CB, Gold AL, Shin LM, Lasko NB, et al. Neurobiological basis of failure to recall extinction memory in posttraumatic stress disorder. Biol Psychiatry. 2009;66(12):1075–82. 19748076 10.1016/j.biopsych.2009.06.026 PMC2787650

[8] Milad MR, Orr SP, Lasko NB, Chang Y, Rauch SL, Pitman RK. Presence and Acquired Origin of reduced Recall for fear extinction in PTSD: results of a Twin Study. J Psychiatr Res. 2008;42(7):515. 18313695 10.1016/j.jpsychires.2008.01.017 PMC2377011

[9] King G, Graham BM, Richardson R. Individual differences in fear relapse. Behav Res Ther. 2018;100:37–43. 29174218 10.1016/j.brat.2017.11.003

[10] Andero R, Brothers SP, Jovanovic T, Chen YT, Salah-Uddin H, Cameron M, et al. Amygdala-dependent fear is regulated by Oprl1 in mice and humans with PTSD. Sci Transl Med. 2013;5(188):188ra73. 23740899 10.1126/scitranslmed.3005656 PMC3732318

[11] Velasco ER, Florido A, Flores, Senabre E, Gomez-Gomez A, Torres A et al. PACAP-PAC1R modulates fear extinction via the ventromedial hypothalamus. Nat Commun, 2022; 13(1). 10.1038/s41467-022-31442-w PMC9334354 35902577

[12] Gilmartin MR, McEchron MD. Single neurons in the medial prefrontal cortex of the rat exhibit tonic and phasic coding during trace fear conditioning. Behav Neurosci. 2005;119(6):1496–510. 16420154 10.1037/0735-7044.119.6.1496

[13] Corcoran KA, Quirk GJ. Activity in prelimbic cortex is necessary for the expression of learned, but not innate, fears. J Neurosci. 2007;27(4):840–4. 17251424 10.1523/JNEUROSCI.5327-06.2007 PMC6672908

[14] Laurent V, Westbrook RF. Inactivation of the infralimbic but not the prelimbic cortex impairs consolidation and retrieval of fear extinction. Learn Mem. 2009;16(9):520–9. 19706835 10.1101/lm.1474609

[15] Burgos-Robles A, Vidal-Gonzalez I, Quirk GJ. Sustained conditioned responses in prelimbic prefrontal neurons are correlated with fear expression and extinction failure. J Neurosci. 2009;29(26):8474–82. 19571138 10.1523/JNEUROSCI.0378-09.2009 PMC2733220

[16] Marek R, Xu L, Sullivan RKP, Sah P. Excitatory connections between the prelimbic and infralimbic medial prefrontal cortex show a role for the prelimbic cortex in fear extinction. Nat Neurosci. 2018;21(5):654–8. 29686260 10.1038/s41593-018-0137-x

[17] Fenton GE, Pollard AK, Halliday DM, Mason R, Bredy TW, Stevenson CW. Persistent prelimbic cortex activity contributes to enhanced learned fear expression in females. Learn Mem. 2014;21(2):55. 24429423 10.1101/lm.033514.113 PMC3895223

[18] Shabel SJ, Wang C, Monk B, Aronson S, Malinow R. Stress transforms lateral habenula reward responses into punishment signals. Proc Natl Acad Sci U S A. 2019;116(25):12488–93. 31152135 10.1073/pnas.1903334116 PMC6589650

[19] Hagihara KM, Bukalo O, Zeller M, Aksoy-Aksel A, Karalis N, Limoges A, et al. Intercalated amygdala clusters orchestrate a switch in fear state. Nature. 2021;594(7863):403–7. 34040259 10.1038/s41586-021-03593-1 PMC8402941

[20] Aschauer DF, Eppler JB, Ewig L, Chambers AR, Pokorny C, Kaschube M et al. Learning-induced biases in the ongoing dynamics of sensory representations predict stimulus generalization. Cell Rep, 2022; 38(6). 10.1016/j.celrep.2022.110340 35139386

[21] Mahan AL, Ressler KJ. Fear conditioning, synaptic plasticity, and the Amygdala: implications for posttraumatic stress disorder. Trends Neurosci. 2012;35(1):24. 21798604 10.1016/j.tins.2011.06.007 PMC3206195

[22] Franklin KBJ, Paxinos G. The mouse brain in stereotaxic coordinates. 2008.

[23] Molina P, Andero R, Armario A. Restraint or immobilization: a comparison of methodologies for restricting free movement in rodents and their potential impact on physiology and behavior. Neurosci Biobehav Rev, 2023;151:105224. 10.1016/j.neubiorev.2023.105224 37156310

[24] Aharoni D, Khakh BS, Silva AJ, Golshani P. All the light that we can see: a new era in miniaturized microscopy. Nat Methods. 2019;16(1):11–3. 30573833 10.1038/s41592-018-0266-x PMC8320687

[25] Zhou P, Resendez SL, Rodriguez-Romaguera J, Jimenez JC, Neufeld SQ, Giovannucci A et al. Efficient and accurate extraction of in vivo calcium signals from microendoscopic video data. Elife, 2018;7:e28728. 10.7554/eLife.28728 PMC5871355 29469809

[26] Pnevmatikakis EA, Giovannucci A, NoRMCorre. An online algorithm for piecewise rigid motion correction of calcium imaging data. J Neurosci Methods. 2017;291:83–94. 28782629 10.1016/j.jneumeth.2017.07.031

[27] Canny J. A Computational Approach to Edge Detection. IEEE Trans Pattern Anal Mach Intell. 1986;PAMI–8(6):679–98. 21869365

[28] Joëls M, Karst H, Krugers HJ, Lucassen PJ. Chronic stress: implications for neuronal morphology, function and neurogenesis. Front Neuroendocrinol. 2007;28(2–3):72–96. 17544065 10.1016/j.yfrne.2007.04.001

[29] Woolley CS. Acute effects of estrogen on neuronal physiology. Annu Rev Pharmacol Toxicol. 2007;47:657–80. 16918306 10.1146/annurev.pharmtox.47.120505.105219

[30] Shansky RM. Sex differences in PTSD resilience and susceptibility: challenges for animal models of fear learning. Neurobiol Stress. 2015;1(1):60. 25729759 10.1016/j.ynstr.2014.09.005 PMC4340080

[31] Cahill L. Why sex matters for neuroscience. Nat Rev Neurosci. 2006;7(6):477–84. 16688123 10.1038/nrn1909

[32] Likhtik E, Paz R. Amygdala-prefrontal interactions in (mal)adaptive learning. Trends Neurosci. 2015;38(3):158–66. 25583269 10.1016/j.tins.2014.12.007 PMC4352381

[33] Herry C, Johansen JP. Encoding of fear learning and memory in distributed neuronal circuits. Nat Neurosci. 2014;17(12):1644–54. 25413091 10.1038/nn.3869

[34] Euston DR, Gruber AJ, McNaughton BL. The role of medial prefrontal cortex in memory and decision making. Neuron. 2012;76(6):1057–70. 23259943 10.1016/j.neuron.2012.12.002 PMC3562704

[35] Svoboda K, Li N. Neural mechanisms of movement planning: motor cortex and beyond. Curr Opin Neurobiol. 2018;49:33–41. 29172091 10.1016/j.conb.2017.10.023

[36] Kim JJ, Jung MW. Neural circuits and mechanisms involved in pavlovian fear conditioning: a critical review. Neurosci Biobehav Rev. 2006;30(2):188–202. 16120461 10.1016/j.neubiorev.2005.06.005 PMC4342048

[37] Arnsten AFT. Stress signalling pathways that impair prefrontal cortex structure and function. Nat Rev Neurosci. 2009;10(6):410. 19455173 10.1038/nrn2648 PMC2907136

[38] Karalis N, Sirota A. Breathing coordinates limbic network dynamics underlying memory consolidation. bioRxiv, 2018;e-location:392530.

