# Supplementary material for: Sex differences in prelimbic cortex calcium dynamics during stress and fear learning
Source: Biol Sex Differ. 2024 Oct 16;15:79. doi: 10.1186/s13293-024-00653-9 (PMC11481719; doi:10.1186/s13293-024-00653-9)
Supplement: Supplementary file 1 — Supplementary Material 1 [file 13293_2024_653_MOESM1_ESM.docx]

Supplementary Material

*
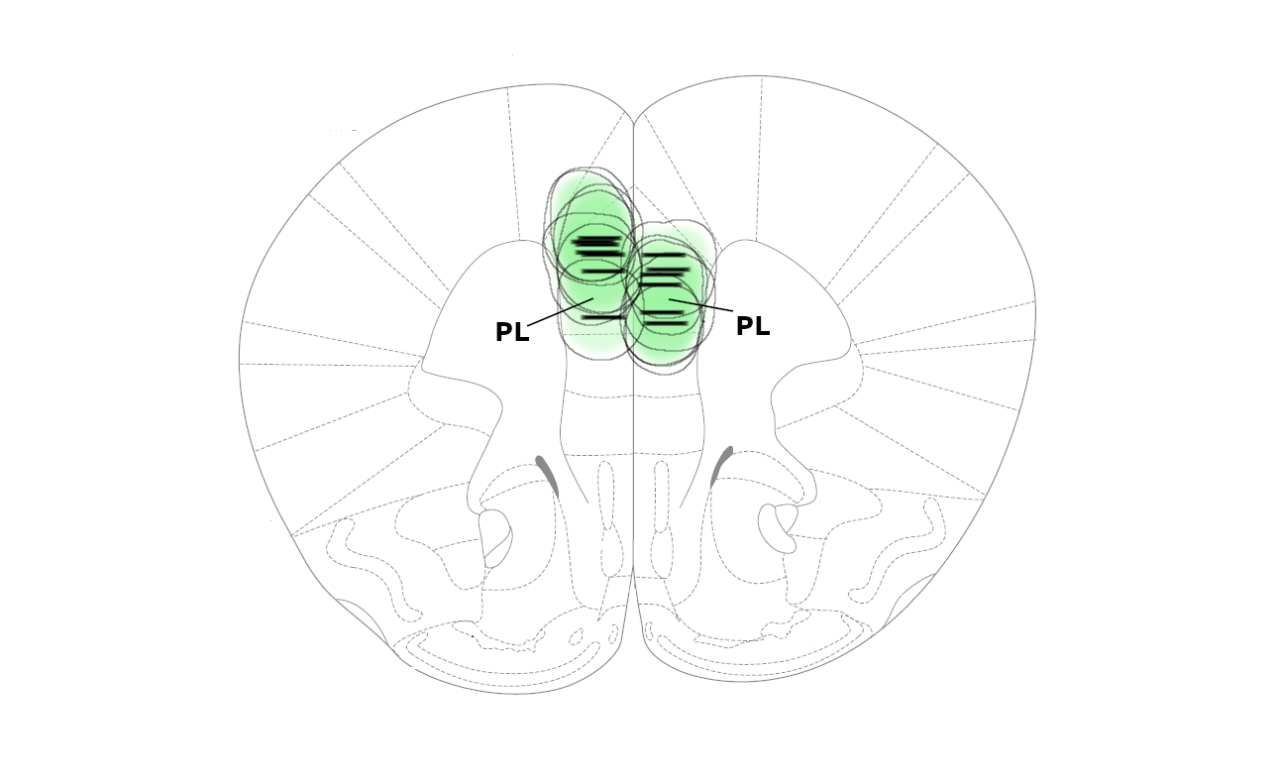
*

*Supplementary Figure 1*: Mouse brain atlas image showing AAV vector spread (outlined green color) and GRIN lens placement (black bars) in the PL region. Stereotaxic coordinates for lens placement: AP + 1.8 mm, LM ± 0.3 mm, DV - 2.3 mm. Adapted from Paxino‘s & Franklin’s Mouse Brain Atlas (2008).

1. $\phi=\frac{TP\cdot TN-FP\cdot FN}{\sqrt{(TP+FP)(TP+FN)(FP+TN)(FN+TN)}}$;
2. $R=\{\phi(n_{1}^{'}),\phi(n_{1}^{'}),...,\phi(n_{m}^{'})\}$;
3. $Z_{T}(n)=\frac{\phi(n)-\overline{R}}{\sigma_{R}}$ .

*Supplementary formulas*: **Formulas used to identify stimulus-responding neurons. A)** Phi Coefficient, φ, used to describe the association between tone presentation and calcium responses. TP and TN are true positives and negatives, and FP and FN are false positives and negatives. **B)** R is a matrix storing all the φ scores computed 1000 (*m*) times for randomized event matrices (*n’*). **C)** Z-score, a statistical measure that quantifies the distance between a data point and the mean of a dataset. This is computed for the original scores with respect to the R matrix.

| **Sex** | **Animal** | **Early IMO** | | **Late IMO** | |
| --- | --- | --- | --- | --- | --- |
|  |  | R Factor | P Value | R Factor | P Value |
| Female | M2 | 0.12900 | 5.8077 E-133 | 0.155307 | 2.71758 E-96 |
| Female | M3 | 0.27684 | 0 | 0.172984 | 2.49106 E-71 |
| Female | M4 | 0.11762 | 6.72283 E-111 | 0.336389 | 0 |
| Female | M6 | 0.08833 | 3.45590 E-63 | 0.424033 | 0 |
| Female | M7 | 0.07559 | 1.05355 E-46 | 0.138972 | 1.49960 E-77 |
| Male | M8 | 0.03345 | 1.78774 E-05 | 0.086686 | 2.84056 E-31 |
| Male | M10 | 0.18348 | 3.98029 E-173 | 0.410467 | 0 |
| Male | M11 | 0.16584 | 2.23557 E-74 | 0.279858 | 4.42500 E-320 |
| Male | M12 | 0.31545 | 0 | 0.415956 | 0 |
| Male | M13 | 0.23247 | 0 | 0.355326 | 0 |

*Supplementary table 1*: **Individual correlation analysis between PL calcium activity (global fluorescence) and movement during the IMO.** The table shows each’s animal R Factor and P-Value for Pearson’s Correlation between global fluorescence and movement in the early and late IMO.

| **Sex** | **Animal** | **FC** | | **FE1** | | **FE2** | |
| --- | --- | --- | --- | --- | --- | --- | --- |
|  |  | R Factor | P Value | R Factor | P Value | R Factor | P Value |
| Female | M2 | - 0.45544 | 3.7942 E-70 | - 0.36679 | 1.5041 E-39 | - 0.41668 | 1.3565 E-51 |
| Female | M3 | - 0.42073 | 4.3873 E-59 | - 0.2956 | 1.2068 E-25 | - 0.29802 | 4.6522 E-26 |
| Female | M4 | - 0.48504 | 1.1975 E-80 | - 0.46733 | 3.9001 E-66 | - 0.26582 | 7.1396 E-21 |
| Female | M5 | - 0.36015 | 1.2113 E-42 | - 0.45724 | 4.3317 E-63 | - 0.08629 | 0.0027645 |
| Female | M7 | - 0.47098 | 4,989E-56 | - 0.21966 | 1.3756 E-14 | - 0.25408 | 3.7832 E-19 |
| Male | M8 | - 0.48793 | 1.1385 E-81 | - 0.59105 | 6,427E-107 | - 0.3367 | 3.2025 E-33 |
| Male | M9 | - 0.45227 | 4.4084 E-69 | - 0.15498 | 6.8195 E-08 | - 0.40711 | 4.0835 E-49 |
| Male | M10 | - 0.4397 | 6.3785 E-65 | - 0.50356 | 3.8078 E-78 | - 0.47192 | 1.4053 E-67 |
| Male | M11 | - 0.46809 | 1.8743 E-74 | - 0.35545 | 4.7095 E-37 | - 0.43602 | 7.3647 E-57 |
| Male | M12 | - 0.52422 | 3.0959 E-96 | - 0.16476 | 9.2975 E-09 | - 0.04131 | 0.15253 |
| Male | M14 | - 0.2929 | 3.9033 E-28 | - 0.27819 | 9.1251 E-23 | - 0.31294 | 1.07 E-28 |

*Supplementary table 2*: **Individual correlation analysis between global fluorescence in the PL and freezing during fear conditioning and extinction.** The table shows each’s animal R Factor and P-Value for the Pearson’s Correlation between global fluorescence and freezing behavior during FC, FE1, and FE2. Animal M12 (Male) was the only exception with no significant correlation (p = 0.15253).
